# Utility of an app-based system to improve feedback following workplace-based assessment

**DOI:** 10.5116/ijme.5910.dc69

**Published:** 2017-05-31

**Authors:** Janet Lefroy, Nicola Roberts, Adrian Molyneux, Maggie Bartlett, Simon Gay, Robert McKinley

**Affiliations:** 1 School of Medicine, Keele University, UK; 2 Medical Education Unit, the University of Nottingham, UK

**Keywords:** Workplace based assessment, undergraduate medical education, feedback culture, technology-enhanced learning

## Abstract

**Objectives:**

To determine whether an app-based software system to support production and storage of assessment feedback summaries makes workplace-based assessment easier for clinical tutors and enhances the educational impact on medical students.

**Methods:**

We monitored our workplace assessor app’s usage by Year 3 to 5 medical students in 2014-15 and conducted focus groups with Year 4 medical students and interviews with clinical tutors who had used the apps. Analysis was by constant comparison using a framework based on elements of van der Vleuten’s utility index.

**Results:**

The app may enhance the content of feedback for students. Using a screen may be distracting if the app is used during feedback discussions.    Educational impact was reduced by students’ perceptions that an easy-to-produce feedback summary is less valuable than one requiring more tutor time and effort. Tutors’ typing, dictation skills and their familiarity with mobile devices varied. This influenced their willingness to use the assessment and feedback mobile app rather than the equivalent web app. Electronic feedback summaries had more real and perceived uses than anticipated both for tutors and students including perceptions that they were for the school rather than the student.

**Conclusions:**

Electronic workplace-based assessment systems can be acceptable to tutors and can make giving detailed written feedback more practical but can interrupt the social interaction required for the feedback conversation. Tutor training and flexible systems will be required to minimise unwanted consequences. The educational impact on both tutors and students of providing pre-formulated advice within the app is worth further study.

## Introduction

Clinical tutors (or preceptors) often have difficulty in providing feedback to their trainees because of time constraints. The design of workplace-based assessment (WBA) tools can result in a greater focus on assessment than on feedback even when the intention is formative.^1^^-^^3^ One way to manage time pressure and to promote constructive feedback is to assist clinical tutors to scaffold learning for their trainees by providing pre-written strategies for use in the event of various possible predicted competency gaps.^4^ The challenge is to present this bank of strategies in a format which is accessible to busy clinicians, enhances the feedback conversation and enables the clinical tutor to add their own ideas. Ideally, a trainee, having had a feedback conversation with their clinical tutor, would be able to reflect on the feedback given, adopt strategies to improve their competence and monitor their own progress with validation and further advice from their clinical tutor.^5^ A written summary of each feedback discussion is not essential but could enhance the value of the assessment by providing an aide memoire for reflection and subsequent discussion, as well as documenting that the process has taken place.

The advent of mobile devices and supporting software systems has made it possible to create “apps” for reference and data-capture uses in almost any setting, including educational assessments. Electronic data capture of mini-CEX assessments via hand-held devices has already been successfully substituted for paper-based assessment of both doctors in training (called “junior doctors” in the UK or “residents” in North America) and medical students, improving timeliness and efficiency.^6^^-^^8^ However, changing the format of an assessment and feedback tool is likely to alter the utility of the assessment in both expected and unexpected ways. For example, the amount of written feedback given decreased when using a hand-held electronic device vs paper-based in a comparative study of mini-CEX assessment of medical students.^9^

The utility of a system of assessment depends upon its reliability, validity, feasibility (or perhaps more appropriately, practicality), acceptability and educational impact.^10^ Assessment in the workplace inherently has low reliability but high validity because it is situated in a real and variable clinical context and within the complexities of a social relationship between trainee and assessor.^11^^,^^12^ It has been argued that the purpose of WBA should therefore be to understand how, why and what trainees are learning rather than attempting to “objectively” or “accurately” measure learning outcomes.^13^ Educational impact should be an additional stated purpose, given the natural suitability of an assessment situated within the learning environment to improve performance.^14^ In studying a system of assessment where the assessment and feedback tool and process are embedded within an app, important questions are whether or not its use is practicable and acceptable for WBA, enhances the feedback conversation and enables learning. For example, mobile devices in the hands of students are not always welcomed by staff and patients in the clinical workplace^15^^,^^16^ yet a small-scale study with medical students in a remedial placement has suggested that student-held devices containing an app for the mini-CEX acted as an ice-breaker in their request for feedback from clinical tutors.^17^

Our study used these “later elements” of van der Vleuten’s Utility Index^10^ as a framework to investigate the utility of an app-based system for WBA and feedback across three clinical years of a medical school programme.

### Research questions:

   •   How practical is it to use an app (mobile or web app) during WBA? (Feasibility)

   •   How acceptable to tutors and students is using an app for WBA and what influence does it have on the feedback process?

   •   What do tutors and students consider is the educational impact of this app-based system of WBA and feedback?

## Methods

### Approach to the study

This study is based in an ongoing action research project to embed and enhance WBA of our students while on clinical placements (or clerkships) at Keele University School of Medicine in the UK. Action research uses mixed methods to triangulate monitoring and evaluation data during programmes of change. This is done rigorously enough to be able to answer research questions and extend general knowledge, as well as solve problems for the local programme.^18^^-^^20^ The monitoring and evaluation activities were approved by the Keele School of Medicine Ethics Committee on 26.1.14.

### Context – WBA developed by action research

At Keele University School of Medicine our WBA programme is entirely formative and does not inform progression decisions. Nevertheless, engagement with the process is mandatory. In primary care (general practice) placements in years 3, 4 and 5 of their undergraduate medical course students consult with patients under supervision and have three WBAs with feedback on their observed consultation skills during each placement. The assessor is the GP tutor who has observed them in practice that week. In secondary care, WBA by observation and feedback is currently optional and is generally done in speciality teaching clinics. Each student therefore has a minimum of 3 WBAs of patient encounters per year.

We have developed our WBA through a series of action research projects.  In one series, we developed an assessment tool (GeCoS – Generic Consultation Skills) which contains the 59 clinical encounter competencies expected of a graduating doctor in nine domains (Opening, History, Examination, Management, Clinical Reasoning, Building and maintaining the relationship, Organisation, Record Keeping and Case Presentation) which now underpin our consultation skills curriculum. The face-validated assessment tool and feedback suggestions are published for others to use. ^4^^,^^21^ We have been using the GeCoS competencies for both formative and summative assessment of consultation skills since 2010. The competencies have not been changed but we have reformatted them in various paper and electronic versions. The set of accompanying strategies for improvement was modified considerably and validated by medical student panels in 2012.^4^

We have also used action research to develop the WBA support systems which contain the GeCoS consultation competencies, the suggestions for how medical students can improve each competency and free text boxes for assessors to remind students about what they did and give additional advice on how to improve. The early iterations of the online WBA system required networked Internet access and generated a utilitarian feedback summary in an unattractive format, which students struggled (or neglected) to read. Despite this, we decided to continue generating written summaries because this seemed to be enhancing the quantity and quality of verbal feedback.^22^  Wishing to improve the feedback system, we postulated that a handheld assessment device which supports audio recording (such as the student’s or assessor’s mobile phone or tablet) should facilitate the dialogue between tutor and student during WBAs. In addition it might be used to capture parts of that dialogue and save time. We considered that a WBA platform that generates a feedback summary as a downloadable PDF should also improve the acceptability of the feedback summary to the student, thereby increasing its utility.

In Cycle 1 (2013-14) of the current Reflect-Plan-Act-Observe action research cycle, we developed, piloted and refined the consultation skills WBA and feedback system. This system comprised a web app and mobile apps for the two predominant mobile communication platforms, along with server-based infrastructure for collecting, processing, analysing and storing the completed assessments. The web app and two mobile apps perform the same function and each contain the GeCoS tool for assessment and feedback but present it differently to suit the format of the device being used. The apps are freely available online and in app stores, but require a Keele log-in to use. We are happy to provide a test student log-in on request.

Cycle 2 (2014-15) involved roll-out of the apps to all year 3, 4 and 5 medical students and their clinical tutors for all WBAs that year. Students received written summaries of the assessments via the School’s online feedback portal.

### Participants

Student participants in this part of the action research project were drawn from year 4 medical students who had experienced formative WBAs over the previous two years in both general practices and hospitals and had thus had experienced the “old” WBA system in their third year and the “new” app-based system in their fourth year. All students on two successive women’s health blocks were invited to participate. These students had all had three WBAs during a four week block in general practice in year 3 and another three WBAs in a four week block in general practice in year 4. Some of them had also had WBAs in hospital teaching clinics in their women’s health block. The students were assessed by numerous GPs, whereas the hospital assessments for these students were conducted by one of the authors (NR). Of the 32 year 4 students invited to two focus groups, 21 participated. Participants of focus group A had their GP block at the start of year 4 while those in focus group B had their GP block half way through the year and shortly before both focus groups were held.

In addition, tutors who had used the app four times or more during the study period were invited by email to consent to a telephone interview. Of 40 clinical tutors invited to interview, 11 volunteered and 10 (23%) were interviewed (7 GPs and 3 hospital doctors). One GP and one hospital doctor among the ten interviewed are authors of this study so their data is not quoted in this paper but their feedback about the app was valuable to the problem-solving side of the action research.

Monitoring data about the usage of both the web and mobile apps, together with the students’ use of the feedback portal are securely stored in a relational database system housed within a Keele-based server. Usage data from all WBAs for year 3, 4 and 5 students was anonymised and monitored for this study.

### Data collection and analysis

We used mixed methods: amalgamating quantitative utilisation data with the qualitative experience data from interview and focus group data.

The medical school’s database was queried to show:

   •   Numbers of WBAs carried out in GP practices and in hospitals.

   •   Numbers of WBA feedback summaries created using the web app and the two mobile apps.

   •   Per-click usage monitoring of the student feedback portal - how many times individual students had accessed their feedback summaries following each WBA.

We conducted two focus group meetings with year 4 medical students. Thirty two students were invited to attend the focus groups, which were facilitated by two research assistants who were not involved in the students’ education. An agreed discussion guide was used in each focus group (Appendix 1).

Telephone interviews with clinical tutors were initially conducted by an independent research assistant. The final four interviews took place after the research assistant’s contract had ended, and were therefore conducted by one of the researchers (JL) using the same interview schedule (Appendix 2).

Focus groups and interviews were audio-recorded and transcribed, and any identifying information removed. Analytic rigor was ensured by two researchers (JL and NR) independently coding the data before testing and achieving consensus. We then used an analysis framework based on the selected elements of van der Vleuten’s utility index^10^ and arranged our codes by constant comparative analysis of all the data that could inform each element.^23^^,^^24^ Using open coding and memo-writing we searched for both expected and unexpected emergent themes in order to develop explanatory theories about the effect of the app-based system on the feedback process and content and, for the purposes of the next cycle of action research, how to improve the process and the technical features of the app.

## Results

**Figure 1:**
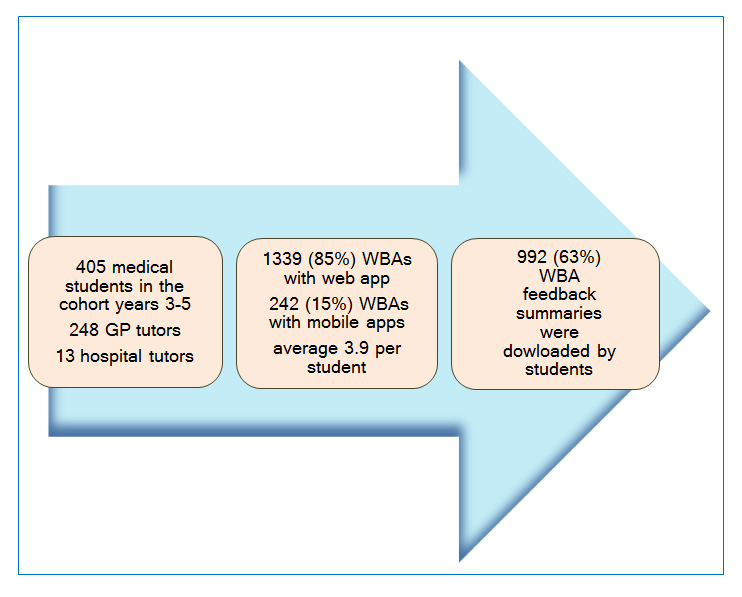
Monitoring data for usage of the new WBA and feedback system 2014-15

In the academic year 2014-15 the Keele Workplace Assessor app was used a total of 1581 times for conducting WBAs of 405 year 3, 4 and 5 students by 261 clinical tutors (248 in GP and 13 piloting it in a hospital setting). Of these, the web app was used 1339 times (85%) and the combined mobile apps were used 242 times (15%). Each tutor used the app between one and twelve times. Students accessed their WBA feedback portal to read their feedback summaries 992 times in the academic year 2014-15 accessing 63% of summaries produced (if each click was to a different summary). These monitoring data are represented in Figure 1.

From the focus groups and tutor interviews we present evidence of influences of the app on the practicability and acceptability of WBA, on the feedback conversation, and perceived educational impact of this app-based system for formative WBA. Under each of these headings there were expected (or intended) and contrasting findings.

### Feasibility of using an app (mobile or web app) during WBA

Table 1 shows examples of student and tutor comments about the accessibility of the mobile or web app.  Students and those tutors who used it considered that the mobile app was easy to download and could be used anywhere; a particular benefit in hospital settings with competition for the available computers, also obviating the need to find the web app. Tutor comments suggested that if not using the same computer each time then the search for the email with the link to the web app made it a struggle to find. Using the mobile app was also found by some to be a faster process than using the web app (Comment 1). However, the vast majority of feedback summaries (85%) were generated using the web app rather than the mobile app. A few (described by students as ‘younger’) GPs were however using the mobile app and were dictating the text of their feedback. Students commented that the mobile phone screen size and typeface are too small. As typing on a mobile phone was not considered practical by students or tutors, it worked well only if the phone had speech recognition. Some tutors used their own tablet devices.

Students commented on the range of tutors’ ability to use mobile devices and navigate the app. They felt that some tutors were looking to them for help implying that they needed further training (Comment 2).

Tutor preference for the web app over the mobile app was unexpected. GP tutors explained that they preferred to type, partly through habit but also because they found that they were more able to think when typing which was helpful when they were trying to craft good feedback (Comment 3). We also infer from the interviews that this is because GPs may not have Wi-Fi or good cellular coverage but all have computers with Internet access. The inaccuracy of voice recognition was also a problem for some (Comment 4).

By contrast, those who expressed a preference for the mobile app found it faster to access and appreciated being able to dictate their free text comments (Comment 5). The mobile app was compared favourably to the web-based mini-CEX and similar WBAs for trainees by a hospital tutor, largely because of the ease of access and use of speech recognition.

The app (both web and mobile) was generally regarded by tutors as a time-saving and effort-saving way of producing feedback (Comment 6) although habits formed on other feedback tools may shape the way this one is used (Comment 7). Although few tutors completed the assessment using the app during student consultations, the majority used it to give an overview feedback summary afterwards (Comment 8).

**Table 1:** Examples of comments about the feasibility/ accessibility of an app for WBA

Comment	
Comment 1	In the hospital it was easier having it on a phone than trying to find an empty monitor. You have to tailor it to the situation if you are with a younger GP or in the hospital where you haven’t got much time you use the app on the phone. If you have got time you use the online version. Student 3(f) Focus Group A
Comment 2	My doctor is an older guy who couldn’t use a phone. He gave it to me and said just tick that. So it wasn’t an assessment. Student 1(m) Focus Group B
Comment 3	I’m used to writing reports …I prefer to reflect a little bit myself and just formulate what it is I want to write down because when I’m giving feedback there’s a lot more communication going on than just verbal feedback… So when you’re writing it in black and white, you have to be a little bit more careful of how you might phrase something. Tutor 5 (GP)
Comment 4	I don’t dictate, because you’re constantly checking the words, I find I actually type faster than I can speak, dictate and check. Tutor 4 (GP)
Comment 5	There’s always that ‘what am I going to say?’ first, you think about it and then you say it. I’m so used to dictating in clinic so I can think what I want to say and it doesn’t faze me. Tutor 10 (H)
Comment 6	You choose your three [strengths or priorities for improvement] which you want to make comment on and it takes you to those and so you’re not having to screen scroll down pages of areas you don’t want to comment upon. Tutor 6 (GP)
Comment 7	There’s still the automatic kind of assumption - I can only really give a short feedback like I normally would. But once you kind of get comfortable to the actual dictation then I think it would help to give more detailed feedback. We give detailed feedback all the time verbally. We just don’t give them any record of it. Tutor 10 (H)
Comment 8	Mine was really good in GP. She wrote everything down in each consultation and then summarised in the GeCoS. Student 2(f) Focus Group A

### Acceptability of the app for WBA and effect on the feedback conversation

Table 2 shows examples of student and tutor comments about the acceptability of the app for WBA, and how it was used in feedback.  The mobile app was designed to be downloaded to the student’s mobile device and handed over by the student when seeking feedback from a tutor. There were students and tutors who voiced discomfort about the student’s mobile phone being used to capture feedback. They indicated that the mobile phone was private and not for others to handle (Comment 9). This had not been anticipated.

Most tutors used the app as a summarising tool for discussion that had already taken place and not as a teaching tool during the discussion. A barrier preventing it from enabling discussion was “screen distraction” - the need to look at a screen to search and type (Comment 10). The analogy of computers in patient consultations was used by tutor 2 who implied that tutors might learn to use the app during the feedback discussion (Comment 11).

Students commented that it was sometimes embarrassing to be present when the feedback was being given, and it might cause the written feedback to be less honest. There was some debate in focus group B about whether they would prefer their feedback summary to be generated by their tutor when they were not present; some suspected the tutors would forget what had been discussed.

Although in its first year the app has been used mainly for mandatory WBAs in GP, it has been piloted in hospital teaching clinics. Student feedback about the impact that had on the feedback obtained was very positive (Comment 12).

**Table 2:** Examples of comments about the acceptability of using an app for workplace assessment and its impact on the feedback conversation

Comment	
Comment 9	Handing over your phone is awkward. Why do the med school expect you to use your (own) phone? Student 4(m) Focus Group A
Comment 10	After looking at them consult I would have given them quite a lot of feedback verbally and then I would use GeCoS to sort of back that up. I might have done it once with them sitting beside me but I found that I’m more concentrating on the computer. I’d rather be concentrating on the student in front of me so I wouldn’t do it that way. It’s an opportunity for them to ask questions. Tutor 5 (GP)
Comment 11	I think you spend more time trying to make the IT work than you do try to make the conversation work, you get distracted by it. It’s a bit like a GP consultation and the role of a computer, the computer is there as an aid and it’s not there to guide the consultation. Patients complain if you spend all the time looking at the screen, so students get frustrated if you’re kind of there texting or whatever it is … the principle of it was brilliant and I am sure it can be made to work a bit better. You know, students live on their phones so that’s great. Tutor 2 (GP)
Comment 12	If we got feedback more often, like in that clinic, like if every placement had something like that, if doctors were more aware of the app it would be so useful for getting feedback, ‘cause it’d just be like right we’re going to do a quick GeCoS now on what you just did, and that’d be great. Student 3(f) Focus Group B

### Educational impact of the app-based GeCoS WBA system

Table 3 shows examples of student and tutor comments about the impact of this WBA system on the content of feedback. Students and tutors appreciated the structure of the GeCoS tool and the guidance provided within the app because it prompted the giving of detailed feedback about both strengths and areas for improvement in a framework that was aligned with their curriculum (Comments 13 and 14).

**Table 3:** Examples of comments about the educational impact of the app-based GeCoS workplace assessment system

Comment	
Comment 13	Within each section there were various descriptors of what you would hope to see in good consulting and those are quite helpful to be able to read through with the student to sort of say these are things that I’m talking about Tutor 5 (GP)
Comment 14	Did you find that your GP people would just tell you anyway? That you didn’t really need the whole formality of (using the app)..?
	Yeah but they’d forget … whereas if you’d got those bullet points in GeCoS they’d think oh actually you’ve done x, y and z really well, but I forgot to tell you about that. Students 2(f) and 3(f) Focus Group B
Comment 15	We have lots of discussions erm and then I suppose one of the difficulties is you’re having discussion and then trying to put that in a structured format within GeCoS.
	Interviewer: So is GeCoS a constraint?
	Yes I suppose in some ways it is but if you didn’t have that structure then other areas of feedback might be missed. I don’t know really. I can’t see it working if you didn’t have some formal assessment tool…. I think one of the downsides of it is that it’s so comprehensive that actually sometimes it’s quite hard to find the slot to put your feedback. Then it gives you a structure because otherwise you may end up just with a bit of waffle in a box which could easily turn into very sort of limited value for the student. Tutor 7 (GP)
Comment 16	I would really like a truthful feedback rather than a tick box thing, nearly a letter from my GP just saying this is what I thought you did really well but this is what you’ve improved on.
	It’s finding the middle ground, isn’t it? Something that will prompt them but doesn’t then limit them It’s so easy for them to tick the box whereas if they have to physically write something, they have to think about what it is and if they can’t think of anything they don’t write anything, it’s as simple as that. Students 4(m), 6(m) and 2(f) Focus Group B

The GeCoS app incorporates suggested text for giving students specific advice on each competency identified as needing improvement. This caused considerable concern to students particularly because they perceived the feedback tool as being too big and too difficult to navigate. Tutors interviewed also mentioned the size as being a problem and some saw the tool as prescriptive but could still see a benefit in terms of the structure and specificity it could give to their feedback which would make it more helpful for the students (Comment 15).

Students suggested that it might be too easy for tutors to tick boxes and that might result in them not thinking enough about what they needed to say, but could see that the app provided helpful guidance (Comment 16).

**Table 4:** Examples of comments about the value of the app in capturing a written record

Comment	
Comment 17	I think it’s a good idea because when I give feedback to students it’s not recorded, I just say what I think and they nod away and they say thank you very much, that was useful, but it’s not recorded in any way…They can’t remember everything I say because it’s quite a lot. Tutor 3(H)
Comment 18	I haven’t even opened the GeCoS feedback emails because I sat down and did it with him so I knew exactly what he said. Student 4 (m) Focus Group B
Comment 19	If you do well in GeCoS it doesn’t seem to matter? And if you do badly what does it matter? What is the point of it then? Student 6 (m) Focus Group B
Comment 20	At the end of the day the tool provides a record and it’s great if the students have a record afterwards so they can go back and look at the points but that is a very small part of the interaction, the main part of the interaction is what goes on as you have that meeting. Tutor 2 (GP)
Comment 21	My GP in 3rd year didn’t tell me when he was doing them and I only found out afterwards and that could have been really bad but I found it really useful because he was really honest. At the time he had told me things, but (the written feedback) was really detailed because he had gone and done it in his own time. Student 5 (f) Focus Group A
Comment 22	Mine was really good. He did a GeCoS with my first consultation and then every GeCoS after that he referred back to it. Student 3 (f) Focus Group A
Comment 23	I think you always get better feedback if you’re not there. They can’t say the negative stuff when you are sat there. Student 2 (f) Focus Group B
Comment 24	There was one that I had erm, maybe it was a bit of a cop-out but he was quite tricky, quite difficult to talk with so I used the GeCoS feedback as a means of being a bit more direct in terms of some advice and feedback for him and then he came back to me on it and we had a much more open discussion about it. So in that sense it actually worked quite well ‘cause once it’s written down on paper he took a bit more notice of it. Tutor 7 (GP)
Comment 25	You feel that somewhere at the top there’s people collating data and they want you to fit into that box. Tutor 6 (GP)

### The value (educational and otherwise) of the app in capturing a written record

Table 4 shows examples of student and tutor comments about the value of creating a written feedback summary. Capturing feedback was seen as worthwhile by tutors (Comment 17), but to most students the written feedback summary was not seen as being of much value compared to the informal feedback discussions and some were unsure of its purpose, believing it to be feedback for the medical school (Comments 18 and 19). This is borne out by triangulation with the usage data from the student feedback portal – one third of feedback summaries were not accessed by students, meaning that they could not have read those written summaries. There was recognition, too, by tutors that the feedback discussion was more important than the written summary (Comment 20). The students who did value the written summary were those who got something more in writing than they had been given face to face, and those who liked to compare one with the next (Comments 21 and 22). One unexpected value of the written feedback mentioned by both students and tutors was that it could be used to say things that were awkward to say face to face (Comments 23 and 24). Also unanticipated and less desirable was that some tutors prepared their feedback summary (because it was submitted electronically) with two recipients in mind – the student and the medical school, which seemed to influence the feedback they gave (Comment 25).

Some tutors also mentioned the value of having their written summaries of feedback to use as evidence of teaching or to remind them about the student if they were later asked by them for a reference.

## Discussion

We studied aspects of the feasibility, acceptability and educational impact of an app-based system to support the production of written summaries of formative WBA for undergraduate medical students. This framework for analysis was used to find both expected and unexpected themes. We expected that the system would enhance the feasibility and acceptability of formative WBA and produce more useful feedback. Unexpected emergent themes were deliberately sought in order to develop our understanding of the impact of new technologies on existing medical education practice.

As expected, tutors perceived both web and mobile versions of the app to be time-efficient and helpful when crafting useful feedback. The requirement to use WBA feedback apps three times in a placement did appear to promote the importance of formative assessment and empower tutors to give detailed and specific feedback. Tutors were confident that they would find appropriate supporting text in the GeCoS tool to help them to create the summaries.

Students were less impressed by the embedded strategies, viewing them as too easily selected and therefore of doubtful personal relevance while recognising the alternative might be to receive less feedback. Students valued highly the free-text feedback provided by their tutors, especially when they perceived that the tutor had put a lot of effort into it. This suggests an unexpected negative feature of an app developed specifically for time-efficiency, if the value placed by students on their feedback is a function of their perception of the effort required to produce it.

Contrary to expectation, we found a preference for the web app amongst GP tutors. Incorporation of the dictation facility in the mobile app was not as appealing as we had expected: those who tried dictating using speech recognition facilities on a mobile device liked the speed but some were concerned about its accuracy. Preference for the web app was explained in three ways: either GP tutors were accustomed to using computers, or they wanted to consider what they committed to writing or they were reluctant to use the student’s device. Tutors who are used to touch screens and speech recognition are starting to use the mobile app in hospital and GP. It may become more acceptable as it becomes more familiar.

As to acceptability, the face-to-face feedback discussions were generally highly valued by students, but some reported that being present when their feedback summary was generated was uncomfortable. This was unexpected and seemed to relate to witnessing their tutor struggle to use the app or waiting passively while they were typing. Some students disliked their mobile phone being used as an educational tool by their tutor, a barrier we had not anticipated.

The app does facilitate learning, but not as envisaged. Although it was designed to enhance the feedback conversation, in the first year of using the app, the majority of tutors have used it at the point of generating feedback summaries rather than using it as a teaching aid during the feedback dialogue. Our previous research suggests that the requirement to create a feedback summary may enhance the feedback dialogue.^22^ In this study, tutors appreciated that the app-based assessment and feedback tool provides structure and curriculum-aligned advice. We suggest that this could influence the feedback dialogue as tutors become familiar with the students’ curriculum through repeated use of the app. Students were less sure than tutors of the utility of the app and only a minority felt that the written feedback summary added value. This was more likely if the student was not present when the written summary was generated.

This study contributes to the literature about the utility of apps in clinical WBA in two ways. Firstly, plurality of platform is important: our provision of both mobile and web apps for the same assessment and feedback tool showed that, given the choice, tutors tend to work on a platform with which they are familiar. This echoes previous comparisons of paper-based and electronic marking, when for example, few used the new electronic assessment system with their trainees.^25^ We have found that it is important that an app-based system is flexible enough to accommodate different experience and skills in typing, dictating and incorporating technology into work and life. This implies that future clinical tutors who have grown up with mobile devices and are “digital natives”^26^ will use technology differently than the current generation of tutors who are mostly “digital immigrants”.^27^

Secondly, the study gives insight into the complexity of introducing an electronic agent into a social interaction, the feedback conversation. Electronic devices in patient consultations such as a mobile device or computer screen become a third and intrusive party.^28^^-^^31^ In the same way, the assessor’s focus on the screen can subvert the feedback discussion, even though it can enhance the content. Training in the use of electronic devices may improve the feedback process, as it has done for the doctor-patient-computer consultation.^32^

### Limitations of this study

Action research is real and messy. It facilitates improvement of a system but findings relevant in one setting must be applied with caution in different contexts. Nevertheless we consider that the lessons we have learned are generalizable to the implementation of other app-based systems in other settings.

While both tutor and student stakeholders evaluated the system, the number of participant tutors was smaller than students. The students in focus group A all had their workplace assessments at the start of the academic year and group B later in the year: this provided some insight into the tutor’s learning curve. Tutors who volunteered to be interviewed may have had stronger positive or negative feelings about the app than those who did not.

Though a change of interviewer, particularly to one who might be perceived to have a vested interest in the outcome of the evaluation (JL), might be viewed as a limitation, there were no obvious differences between the resultant themes of the interviews by the independent researcher and those interviewed later by JL, although the interviewees did ask questions of JL about the app.  The study was conducted in a single school with a ‘bespoke’ solution to its assessment support.

### Implications for practice and next steps

Multiple interfaces are needed when setting up a system of electronic WBA. Mobile and web apps suit different environments (with varied computer and Wi-Fi availability) and people (with varied competencies). While the dictation facilitation was useful, some tutors prefer to type their feedback. In setting up such a system, it is worth profiling the users for their familiarity not only with mobile technology, but also being a “native” at typing or dictation in their daily work, as such preferences are strong.

A written summary has multiple roles and students are not the only beneficiary of their feedback discussion being captured in writing: it also benefits tutors by educating them about the students’ curriculum, evidencing their teaching and aiding their recall for the future.

Co-completion of the summary of a feedback discussion has advantages and disadvantages. Although feedback should be a conversation between student and tutor, the incentive to read a summary of that conversation may be removed by the feeling that it contains nothing unknown.

Provision of preformed strategies for improving clinical skills has a downside as well as benefits. The ease of selection, which is an attractive feature to tutors, makes students suspicious of their personal relevance because convenience may have bypassed thought. This broadens the canvas for staff training to include information-sharing skills for formative assessors and how to use standardised materials in a customised fashion.

In conclusion, medical educators are adapting to the digital era but interactions with learning and assessment systems risk interrupting the inherent social interactions in education. Continued successful integration of technology in medical education will require carefully planned training and mentoring and systems sufficiently flexible to cope with the subtle demands placed upon them.

### Acknowledgements

We are grateful to the students and clinical tutors who participated in interviews and focus groups, to Domenica Gentile for interviewing tutors and for facilitating student focus groups with Christalla Pithara and James Archer. We are also indebted to Muhammad Gul for his systematic review of the literature in this area, conducted as his MMedEd project. Transcription costs were covered by Keele University School of Medicine.

### Conflict of Interest

The authors declare that they have no conflict of interest.

## Supplementary material

Supplementary file 1 Appendix 1. Focus Group Facilitator Guide

Supplementary file 2 Appendix 2. Guide for Telephone Interviews of Clinical Tutors
