# Supplementary material for: Utility of an app-based system to improve feedback following workplace-based assessment
Source: Int J Med Educ. 2017 May 31;8:207–16. doi: 10.5116/ijme.5910.dc69 (PMC5457783; doi:10.5116/ijme.5910.dc69)
Supplement: Supplementary file 1 — Appendix 1. Focus Group Facilitator Guide [file ijme-8-207-S1.pdf]

Appendix 1

Focus Group Facilitator Guide

INTRODUCTORY QUESTIONS

What do you understand by WBA?
What are your thoughts about WBA?

TOPICS FOR EXPLORATION

1. **General exploration of the app**
   - Have you made use of the WBA app?
   - Thoughts on the app?
     - Ease of use?
     - Ease of access?
     - Quality of the app?
   - How is the app being used?
     - When is it accessed?
     - How often is the app accessed?
     - Is it used as a resource or repository?
     - Is text dictated or typed?

2. **Usefulness of the app to students**
   - Is the app more useful than the previous online method?
     - If so, how and why is the app more useful?
   - Does the app improve the quality of feedback
   - How could the app be made more useful?

3. **Outcomes of the WBA**
   - What do students do with the feedback they receive?
     - Ignored or acted upon?
     - Value placed upon the feedback?
     - Does the app influence how the feedback is used?

Conclusion:

- Are there any additional comments anyone would like to make regarding our discussion around WBA?
