# Supplementary material for: Utility of an app-based system to improve feedback following workplace-based assessment
Source: Int J Med Educ. 2017 May 31;8:207–16. doi: 10.5116/ijme.5910.dc69 (PMC5457783; doi:10.5116/ijme.5910.dc69)
Supplement: Supplementary file 2 — Appendix 2. Guide for Telephone Interviews of Clinical Tutors [file ijme-8-207-S2.pdf]

Appendix 2

Guide for Telephone Interviews of Clinical Tutors

QUESTIONS

1. **What do you think of your workplace based assessments using the new GeCoS or LCAT app**

   - ask for any other positive and negative views after they have said what they want to
   - ask how many GeCoS assessments they have had last year and this year so far

     - ➢ Thoughts on the app
       - User-friendly?
       - Ease of access?
     - ➢ How is the app being used?
       - When is it accessed?
       - Whose device is being used (the student's or the assessor's? mobile device or computer)
       - Is it used as a resource for feedback content or as a repository after the feedback discussion?
       - Is text dictated or typed?

   Usefulness of the app to students

     - ➢ How does feedback with the GeCoS (and LCAT) app compare with the previous online process?
     - ➢ Does the app improve the quality of feedback?
     - ➢ How could the app be made more useful?

2. **Do you think using the app altered the content of the feedback you give compared to informal feedback discussions? (Quantity? Detail? Alignment with curriculum?)**

3. **Any other comments?**
